# Method of Individual Adjustment for 3D CT Analysis: Linear Measurement

**DOI:** 10.1155/2016/6893072

**Published:** 2016-12-13

**Authors:** Dong Kyu Kim, Dong Hun Choi, Jeong Woo Lee, Jung Dug Yang, Ho Yun Chung, Byung Chae Cho, Kang Young Choi

**Affiliations:** Department of Plastic and Reconstructive Surgery, School of Medicine, Kyungpook National University, Daegu, Republic of Korea

## Abstract

*Introduction*. We aim to regularize measurement values in three-dimensional (3D) computed tomography (CT) reconstructed images for higher-precision 3D analysis, focusing on length-based 3D cephalometric examinations.* Methods*. We measure the linear distances between points on different skull models using Vernier calipers (real values). We use 10 differently tilted CT scans for 3D CT reconstruction of the models and measure the same linear distances from the picture archiving and communication system (PACS). In both cases, each measurement is performed three times by three doctors, yielding nine measurements. The real values are compared with the PACS values. Each PACS measurement is revised based on the display field of view (DFOV) values and compared with the real values.* Results*. The real values and the PACS measurement changes according to tilt value have no significant correlations (*p* > 0.05). However, significant correlations appear between the real values and DFOV-adjusted PACS measurements (*p* < 0.001). Hence, we obtain a correlation expression that can yield real physical values from PACS measurements. The DFOV value intervals for various age groups are also verified.* Conclusion*. Precise confirmation of individual preoperative length and precise analysis of postoperative improvements through 3D analysis is possible, which is helpful for facial-bone-surgery symmetry correction.

## 1. Introduction

Computed tomography (CT) is an imaging technology that allows cross-sectional imaging of a subject to be conducted using a rotating X-ray tube and detector, with the aid of a computer. This technology can be used to record and visualize tomographic images of the body, which is not possible via general photography. Further, two-dimensional data can be used to construct a three-dimensional (3D) image [1].

3D CT magnification power is not site dependent, and important sites can be observed in detail without image overlapping [2]. Further, 3D CT yields high-contrast resolution that allows discrimination between tissues with physical density contrasts of less than 1%, unlike previous radiography techniques that could only discriminate between tissues when there was a minimum density contrast of 10% [3]. The 3D images are obtained via reconstruction using a number of individual images, and various imaging techniques, such as multiplanar reformatting, surface rendering, and volume rendering, can be applied during this process. Further, areas of interest can easily be assessed from various perspectives, as image rotation is possible. 3D CT has a number of advantages over radiographs that render 3D structures into two dimensions, for example, front cephalometric radiographs; however, in various respects, front cephalometric radiographs provide more accessible diagnostic data [2, 4].

Recently, the medical field has been attempting to overcome the limits of two-dimensional (2D) diagnosis through use of 3D CT. Previous diagnoses using cephalometric radiographs were performed by projecting a 3D structure onto a 2D plane, which leads to distortions in length, angle, and form. Thus, inaccurate results are obtained from quantitative evaluations of these images. Further, cephalometric radiography can measure side landmarks only; thus, it is difficult to assess problems like facial area asymmetry.

Accordingly, a number of studies based on 3D CT reconstructed images have been published. However, no studies have been conducted on the variation in the quantitative length measurements for each CT image employed in 3D reconstruction. For example, changes in the tilt and display field of view (DFOV) values cause irregular changes in the length values measured from the resultant images. As a result, researchers believe that there is a significant correlation between the lengths apparent in the 3D reconstructed images and the physical measurements, which can be determined if the DFOV and tilt values (among the various parameters) are used to revise the values. This approach is feasible, as significant correlation has already been noted in quantitative angle analysis of 3D CT reconstructed images, and it would be useful if some relationship was found between 3D CT measurements and the corresponding physical values [5].

Therefore, in this study, we perform quantitative measurement of four different types of dry skull models, using length measurements between different points on the skulls, and aim to develop a method that facilitates higher-precision measurement from reconstructed images for higher-accuracy 3D analysis.

## 2. Methods

**Figure 1:**
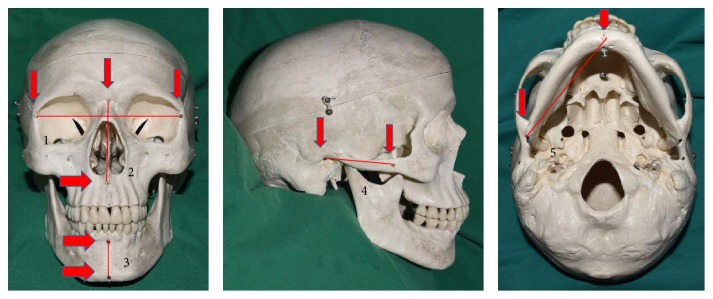
Skull models and measurement points. 1.3 mm titanium screws were fixed onto four differently sized skull models, and five linear distances between N, A, B, Pg, Po, Go, Me, Or, and Ol were set for measurement. 1: Hh; 2: N–A; 3: B–Pg; 4: Po–zygomatic suture; 5: Go–Me.

**Figure 2:**
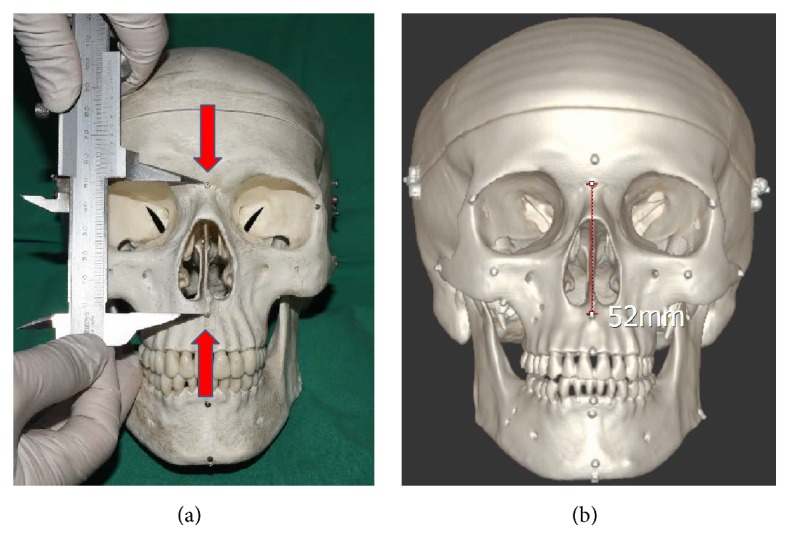
Physical and CT-based measurements of skull models. (a) The five set linear distances were measured using Vernier calipers (real values). (b) The values of the same distances were measured based on the PACS, which was obtained via 3D reconstruction of CT scans (PACS measurement).

We prepared four differently sized dry skull models (three adult and one child size). We then fixed 1.3 mm titanium screws to various points, as shown in Figure 1. Specifically, screws were fixed to the nasion (N), point A (A), basion (B), pogonion (Pg), porion (Po), gonion (Go), menton (Me), orbitale right and left (Or and Ol, resp.), and zygomatic suture. Then, we set a total of five cephalometric measurement intervals, as follows: horizontal reference line, Or–Ol (Hh); N–A; B–Pg; Po–zygomatic suture; and Go–Me. Then, we measured the linear distances between those points using Vernier calipers, as shown in Figure 2. These measurements were the real physical values, hereafter referred to as “real values.” Three different doctors performed each measurement three times; thus, we obtained a total of nine measurements for use in the statistical analysis.

**Figure 3:**
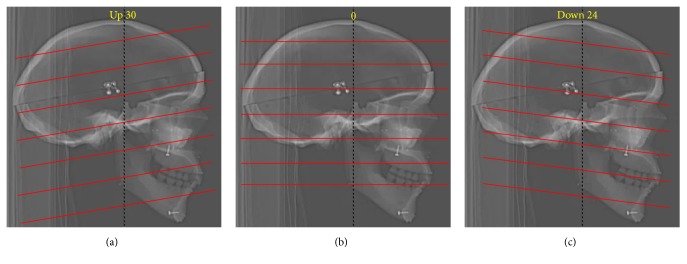
The CT machine was tilted from superior 30° to inferior 24° in 6° increments, yielding 10 CT scans with different tilts for 3D reconstruction. The red lines in the figures indicate the angles at which the CT X-ray beams irradiated the model after tilting. The images indicate the CT scan X-ray beam irradiation angles for (a) superior 30° tilt, (b) 0° tilt, and (c) inferior 24° tilt.

Then, we performed CT scanning of the skull model. We tilted the CT machine from superior 30° to inferior 24° in 6° increments, so as to obtain 10 differently tilted CT scans for 3D reconstruction. Then, we measured the values of the same intervals considered for the “real values,” using the screws visible on the picture archiving and communication system (PACS; Figure 2(b)). As in the case of the real physical measurements, three different doctors performed each measurement three times, yielding a total of nine measurements (Figure 3).

We then compared the measured real values with the PACS-based measurements for the 3D reconstructed images obtained at the 10 different tilt values. In addition, we adjusted the PACS measurement results by considering the display field of view (DFOV) value visible on the 3D reconstruction monitor. We then compared the DFOV-adjusted PACS results with the measured real values. We compared and statistically analyzed each of the values obtained from each of the four models.

Note that we employed a spiral CT (light-speed CT, GE Healthcare, USA) for the CT scans, and the obtained images were reconstructed using the AW Volume Share 5 (GE Healthcare) software, which is a 3D reconstruction program. We utilized IBM SPSS version 22 to conduct Friedman's testing and multiple regression analysis.

## 3. Results

### 3.1. Comparison of PACS Measurements and Real Values on Absolute Difference

We measured the absolute error and percentage error in all planes to compare the absolute values between real values and PACS measurements. In all comparisons, real values were measured more highly than PACS measurements and this was not statistically significant (Table 1).

Real values appeared differently from PACS measurements in all planes and the greatest mean difference was 3.93 mm (8.333%) in P0~zygomatic suture. The difference between real values and PACS measurements did not exceed 1 mm in B~Pg, N~A, and orbital wall side to side (60%) and was measured over 2 mm (2~4 mm) in G0~Me and P0~zygomatic suture (40%).

### 3.2. Comparison of PACS Measurements and Real Values Incorporating Tilt Variations

We tried to figure it out that the changes in the PACS measurements in accordance with the tilt change show regularity on real value. The real values, PACS measurements, and DFOV-adjusted PACS measurements are listed in Tables 2 [Table tab3]–4, respectively. The PACS measurements obtained for different tilts exhibited irregular variations (Table 2) that were within the accepted error range, when compared to the real values obtained for the regular 6° tilt adjustments (Table 3). In addition, the significance probability of the PACS measurement according to the tilt was 0.09, as indicated by Friedman's test of the PACS measurements for various tilts. Hence, we did not obtain a statistically significant result, considering a significance level of 0.05 (Table 5). This result indicates that there is no correlation between the PACS measurements and real values in accordance with changes in tilt.

### 3.3. Comparison between DFOV-Adjusted PACS Measurements and Real Values

**Figure 4:**
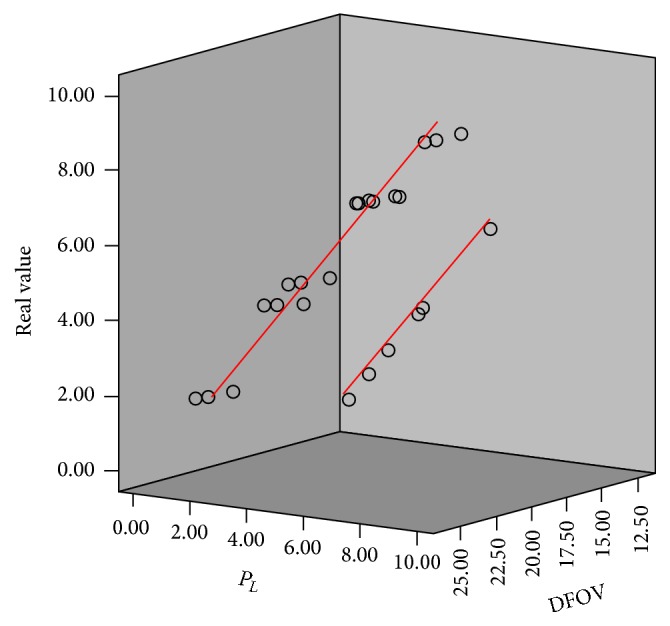
Comparison and linear fitting of DFOV-adjusted PACS measurements and real values.

We performed multiple regression analysis to determine whether or not a relationship exists between the DFOV-adjusted PACS measurements and the real values in all planes. If a relationship was indeed identified, we also aimed to characterize this correlation. The significance probability was less than 0.001, which was significant for a significance level of 0.05. Hence, we obtained significant results. In particular, the PACS measurement at 0° tilt (*t*-value = 73.154, *p* value < 0.001) had a significant correlation with the real value (Table 6). Hence, we confirmed the significance of the relationship between the DFOV-adjusted PACS measurement for 0° tilt and the real value, and we identified the expression required to obtain real length values from PACS measurements. Specifically,

(1) Real  valuemm=0.962×PL+0.045×DFOV−0.583,

where *P*_*L*_ indicates the PACS measurement at 0° tilt (mm). A graph of this relationship is shown in Figure 4.

Using the above method, we performed an analysis to verify whether or not significant results could be found by comparing the measurements obtained for each measurement interval. All of the measurement intervals (Hh, N–A, B–Pg, Po–zygomatic suture, and Go–Me) exhibited statistically significant relations, as shown in the total value analysis (Table 7).

### 3.4. Statistics according to DFOV-Adjusted PACS Measurements Considering Tilt Variations

Based on the above results, we found that the DFOV-adjusted PACS measurements for 0° tilt had a significant correlation with the real values. Therefore, we performed additional analyses to verify the regularity and correlation significance of the DFOV-adjusted PACS measurements for the other tilt values. We verified the variations in the DFOV values in accordance with the changes in tilt for each 3D CT scan image and incorporated the DFOV-adjusted PACS measurements for each tilt angle (from superior 30° to inferior 24° in 6° intervals) using (1), which was obtained for 0° tilt. From the analysis of the DFOV-adjusted PACS measurements, the significance probability for the mean difference in the DFOV-adjusted PACS measurements according to tilt was 0.06, which was statistically insignificant for a 0.05 significance level (Table 8).

As a result of this statistical analysis, we found that the PACS measurements exhibit a significant correlation with the real values when adjusted based on the DFOV values at 0° tilt; however, the changes in the PACS measurements in accordance with the various tilt values were not seen regularity, not significant, even though we adjust PACS measurements with DFOV.

### 3.5. Standard DFOV Values for Various Patient Ages

Based on the above results, we searched for DFOV values that could be used as standard adjustment values for various patient age groups, for a set of 200 patients of various ages who underwent 3D facial CT. We performed the measurements by dividing the patients into three groups, that is, 0–8, 9–15, and >16 years, and examining their 3D CT scans. The results (range of DFOV) are listed in Table 9.

The DFOV value was seen to change in order to allow convenient viewing of the 3D reconstructed image depending on the subject skull size. We also obtained the Korean standard DFOV (KSDOV) values for each age group. These results are useful, as it is possible for different hospitals to share certain patient information without considering the issue of length changes in CT, provided that an appropriate DFOV value is set as a standard for the CT scans (Table 10).

## 4. Discussion

Recently, the importance of quantitative evaluation in 3D CT has increased in accordance with the increased interest in preoperative planning and postoperative result analysis using this technique.

Varghese et al.'s research also presented that the accuracy of quantitative evaluation is important [6], and linear measurement obtained from quantitative evaluation has been used a lot in the field of cranial vault, brain, and spinal cord [7, 8]. When it comes to orthodontics and maxillofacial surgery, exact measurement is an important key point especially in relation to complex craniofacial disorders [6], and there are many studies describing the accuracy of linear measurements, but they are limited to comparing absolute values, yet [9, 10]. Thus, we tried to find the regular results through comparison with the absolute value and the relative value on linear measurement in this study.

The preexisting 2D-image analysis techniques have been helpful as regards preoperative planning and postoperative result analysis; however, disadvantages such as difficulty in measurement point differentiation due to structure overlapping or inappropriate scanning technology have been identified, along with a lack of analysis accuracy and reliability due to image distortion or enlargement. In order to overcome these problems, 3D imaging using CT has been developed. This technique has the advantage of zero site-based magnification errors and a lack of image overlapping, which facilitates detailed observation [11–14].

At present, the data acquired through CT can be reconstructed via volume rendering into a 3D image [15, 16]. Unlike surface rendering, which cannot detect stair-step artifacts or the interior of the bone cortex, this approach facilitates imaging of the cortex interior and 3D observation and measurement. Further, 3D models established using this method can be rotated around the *x*-, *y*-, and *z*-axis, enabling more convenient analysis of the interior of the human body [17, 18].

In order to measure length or area in CT, the number of pixels in the measured image is counted. Measurements close to the real physical values can be obtained using PixelSpacing information stored in the DICOM Tag (0028,0030), along with image-calculated parameter information. This information can be calculated directly from the monitor in sectional view. However, measuring length for a 3D reconstructed model is a different scenario. The images used in 3D reconstruction are simply screen captures transmitted to PACS and are actually 2D images created by including the minimum information necessary for the 3D image without that necessary for measurements, such as the DICOM Tag (0028,0030) PixelSpacing, monitor resolution, magnification, angle value, and *z*-axis value. Therefore, it is generally difficult to gain the absolute value of a real measurement in the sectional view of a 3D image, for example. Further, it is also impossible to compare differences between individuals [19, 20]. Of course it is possible for a relative comparison of perioperative changes or variations in internal organ size to be performed for one individual; however, such an analysis merely shows the rate of change rather than the changes in the absolute values.

Many studies have shown that 3D CT cephalometric landmarks have good reliability, but a large number of variables have been employed to obtain images in those studies. These variables were introduced via the experimental methods, software, visualization method, and anatomical reference selection. Although some studies have used CT and CT datasets to identify cephalometric landmarks for 3D volumetric reconstruction, linear measurement based on 3D volumetric reconstruction of CT data using software still lacks viability and accuracy.

When conducting a study based on a 3D reconstruction model, we can obtain real measurements based on relative comparisons for individuals, and we can define the absolute values if various conditions are set in advance and standardized per individual. First, we must standardize the physical values of the CT scanner during the data acquisition scan. This includes the DFOV, angle value, *z*-axis value, which is a necessary measurement, and the monitor resolution and magnification on the PACS. Once these standardizations have been imposed, we can reconstruct the image in the display window. Then, we can mark the size measurements and transmit the captured screen to the PACS [20]. In such cases, the linear and angular measurements are similar to the real values to a certain degree, and the significance of the correlation between the angular measurement and the real value can be verified. This finding has been reported previously by Oh et al. [21], in their study of conventional lateral cephalometry and 3D CT image reconstruction.

Related to linear measurement, a large number of evaluations had been implemented to verify accuracy and investigate measurement points of 3D CT images. Cavalcanti and Vannier presented the difference between actual measurement of cranial bone and measurement of 3D CT images that is less than 2 mm in most of cases [12]. And Pinsky et al. reported the accuracy of CT images by comparing the actual measurement and the measurement of CT images [22]. Periago et al. also reported the accuracy of linear measurement in craniofacial study, so that those could be useful results clinically [10].

**Figure 5:**
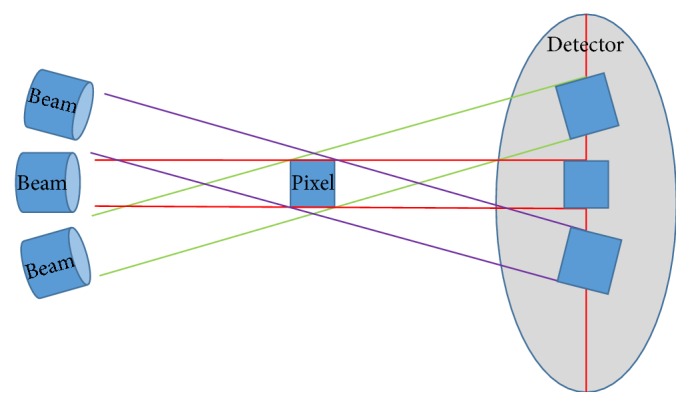
Sizes and shapes of pixel size variations during CT scanning in response to tilt variations.

In this study, we examined whether or not regularized results could be obtained through comparison of linear measurements, similar to the previously examined case of angular measurement. We used the DFOV and tilt values to standardize the physical values of the CT scanner and confirmed the changes in the measurements induced by adjustments of the tilt values. Note that we assumed the changes in tilt value would not affect the angular or linear measurements, as all points of the bone move in the same direction. However, we could not verify regular changes in the measurements in response to the variations in the tilt value when the DFOV was fixed. This can be found from the “Accuracy of Computerized Tomography for the Evaluation of Mandibular Sites prior to Implant Placement” reported by Sforza et al. As revealed in this report, our study could also not observe constant changes depending on tilt values [23]. On the contrary, when the tilt values were fixed and the DFOV values were used for revision, the comparison between the PACS measurements and the real values showed significant correlation; these findings in the linear measurement case are similar to those previously reported for the angular measurement case.  Figure 5 is an illustration showing the effects of tilt changes on the CT scan size; it is apparent that the CT scan size determined by the X-ray beams projected onto the fixed pixels changes in response to variations in the tilt. Hence, the total area of the 2D plane that is initially CT scanned varies in accordance with the change in tilt. Further, variations in the scan image length are induced by this change in plane. We believe that these variations cause the irregularities in the 3D reconstructions based on such 2D scans. However, this issue can be solved by fixing the tilt value during the CT scan. That is, regularized linear measurement values can be obtained by fixing the tilt and DFOV values.

This study has focused on length-based 3D cephalometric analysis using 3D CT, which has not been studied previously. We have found that precise individual preoperative length confirmation and precise analysis of postoperative improvements can be performed via 3D analysis, which will also be very helpful for improving symmetry revision in facial bone surgery. However, this study is limited by that fact that only a small number of measurements were conducted for only four skulls. This limitation was alleviated by the use of several measurements for each case, which were obtained by different doctors. However, the results could be improved by increasing the number of subjects, so as to overcome statistical errors.

## 5. Conclusion

In conclusion, it is difficult to compare absolute values when examining measurements based on 3D CT images, as there are a number of variable factors. Therefore, the various physical factors and measurements must be standardized in order to facilitate accurate analysis. Unlike 2D cephalometry, conventional 3D CT can yield absolute values for angular measurements, but it is difficult to achieve the same accuracy for linear measurements. However, approximate values can be obtained, while more accurate values can be recorded if the image scanning and capturing method is standardized in every individual. In this study, actual length values and those obtained from 3D reconstructed images were compared, considering the tilt and DFOV values. Hence, we confirmed that a relationship exists between the image length as measured from the PACS and the real, physical value. Further, this relationship can be determined based on comparison of the DFOV values when the tilt value is fixed. While the PACS-image length cannot constitute an absolute value, we believe that the absolute value can be inferred through DFOV-value-based revision of each CT image. In addition, DFOV standards for patients can be defined from the KSDFOV values obtained in this study, and the same CT scan information for a given patient can be used in any hospital.

## Figures and Tables

**Table 1:** Comparison of absolute and percentage differences for linear measurements between real values and PACS measurements.

Plane	Measurement	Absolute error				Significance		Percentage error				Significance	
		Mean Dif	SD	95% confidence interval				Mean Dif	SD	95% confidence interval			
				Lower	Upper	*t*	*p* value			Lower	Upper	*t*	*p* value
G0-Me (Lt.)	1	−0.315	0.275	−0.752	0.122	−2.293	0.106	−3.716	4.072	−10.2	2.763	−1.825	0.165
	2	−0.305	0.258	−0.715	0.105	−2.367	0.099	−3.638	3.788	−9.666	2.39	−1.921	0.151
	3	−0.3	0.214	−0.641	0.041	−2.798	0.068	−3.896	2.671	−8.146	0.354	−2.917	0.062

G0-Me (Rt.)	1	−0.218	0.161	−0.474	0.039	−2.703	0.074	−2.829	1.973	−5.968	0.31	−2.868	0.064
	2	−0.198	0.242	−0.582	0.187	−1.634	0.201	−1.845	4.395	−8.839	5.149	−0.839	0.463
	3	−0.215	0.232	−0.584	0.154	−1.854	0.161	−2.365	3.625	−8.133	3.403	−1.305	0.283

B~Pg	1	−0.015	0.257	−0.394	0.424	−0.117	0.914	0.585	12.736	−19.68	20.85	0.092	0.933
	2	−0.033	0.286	−0.422	0.487	−0.227	0.835	5.105	16.844	−21.7	31.908	0.606	0.587
	3	−0.013	0.293	−0.454	0.479	−0.085	0.937	3.677	16.604	−22.74	30.097	0.443	0.688

N~A	1	−0.103	0.212	−0.44	0.235	−0.965	0.406	−1.754	4.063	−8.218	4.711	−0.863	0.451
	2	−0.08	0.291	−0.543	0.383	−0.55	0.621	−0.522	6.516	−10.89	9.846	−0.16	0.883
	3	−0.095	0.272	−0.527	0.337	−0.7	0.535	−1.225	5.484	−9.952	7.502	−0.447	0.685

Orbital wall side to side	1	−0.088	0.092	−0.059	0.234	−1.899	0.154	0.868	1.01	−0.739	2.474	1.719	0.184
	2	−0.06	0.067	−0.046	0.166	−1.796	0.17	0.656	0.663	−0.4	1.712	1.978	0.142
	3	−0.085	0.044	−0.016	0.154	−3.9	0.03	0.969	0.386	0.356	1.583	5.026	0.015

P0~zygomatic suture	1	−0.393	0.299	−0.869	0.084	−2.623	0.079	−8.333	6.283	−18.33	1.666	−2.652	0.077
	2	−0.363	0.391	−0.984	0.259	−1.856	0.161	−5.258	12.945	−25.86	15.34	−0.812	0.476
	3	−0.388	0.353	−0.949	0.174	−2.197	0.115	−7.085	9.543	−22.27	8.101	−1.485	0.234

Absolute error = PACS measurements − real values.

Percentage error = (PACS measurements − real values)/real values × 100.

**Table 2:** Real measurements at each measurement point (real values).

	Skull A	Skull B	Skull C	Skull D
G0~Me (Lt.)	7.76 ± 0.03	7.82 ± 0.03	7.84 ± 0.03	3.33 ± 0.03
G0~Me (Rt.)	7.76 ± 0.02	7.82 ± 0.02	7.84 ± 0.01	3.14 ± 0.05
B~Pg	1.98 ± 0.02	2.02 ± 0.01	2.05 ± 0.02	0.64 ± 0.03
N~A	5.34 ± 0.01	5.39 ± 0.02	5.42 ± 0.03	2.09 ± 0.01
Or~Ol	9.66 ± 0.01	9.69 ± 0.01	9.69 ± 0.01	5.68 ± 0.01
P0~zygomatic suture	4.58 ± 0.01	4.72 ± 0.02	4.77 ± 0.01	1.39 ± 0.01

(Mean ± standard deviation).

**Table 3 tab3:** Mean values of PACS measurements between each cephalometric measurement point.

	Skull A	Skull B	Skull C	Skull D
Superior 30′ tilt	5.75 ± 0.03	5.70 ± 0.03	5.41 ± 0.04	2.68 ± 0.04
Superior 24′ tilt	5.87 ± 0.04	5.80 ± 0.02	5.57 ± 0.04	2.63 ± 0.04
Superior 18′ tilt	5.96 ± 0.04	5.89 ± 0.03	5.71 ± 0.04	2.76 ± 0.05
Superior 12′ tilt	6.07 ± 0.03	6.02 ± 0.03	5.84 ± 0.04	2.70 ± 0.04
Superior 6′ tilt	6.08 ± 0.05	6.13 ± 0.03	5.97 ± 0.05	6.05 ± 0.05
Tilt 0	6.07 ± 0.02	6.10 ± 0.03	5.96 ± 0.03	2.77 ± 0.05
Inferior 6′ tilt	5.74 ± 0.03	5.76 ± 0.03	5.96 ± 0.03	2.73 ± 0.05
Inferior 12′ tilt	5.87 ± 0.02	5.88 ± 0.03	5.98 ± 0.04	2.86 ± 0.03
Inferior 18′ tilt	6.01 ± 0.04	5.97 ± 0.03	6.12 ± 0.04	2.65 ± 0.03
Inferior 24′ tilt	6.09 ± 0.03	6.18 ± 0.04	6.10 ± 0.05	2.69 ± 0.03

(Mean ± standard deviation).

**Table 4:** Mean values of PACS measurements between each cephalometric measurement point, based on DFOV.

	Skull A	Skull B	Skull C	Skull D
Superior 30′ tilt	5.87 ± 0.03	5.85 ± 0.03	5.87 ± 0.04	2.73 ± 0.04
Superior 24′ tilt	5.90 ± 0.04	5.89 ± 0.02	5.89 ± 0.04	2.73 ± 0.04
Superior 18′ tilt	5.93 ± 0.04	5.92 ± 0.03	5.93 ± 0.04	2.73 ± 0.05
Superior 12′ tilt	5.96 ± 0.03	5.94 ± 0.03	5.95 ± 0.04	2.73 ± 0.04
Superior 6′ tilt	5.98 ± 0.05	5.98 ± 0.03	5.98 ± 0.05	2.78 ± 0.05
Tilt 0	6.00 ± 0.02	6.01 ± 0.03	6.01 ± 0.03	2.79 ± 0.05
Inferior 6′ tilt	5.91 ± 0.03	5.90 ± 0.03	5.96 ± 0.04	2.74 ± 0.05
Inferior 12′ tilt	5.91 ± 0.02	5.88 ± 0.03	5.90 ± 0.04	2.73 ± 0.04
Inferior 18′ tilt	5.91 ± 0.04	5.84 ± 0.03	5.86 ± 0.04	2.73 ± 0.04
Inferior 24′ tilt	5.91 ± 0.03	5.82 ± 0.04	5.83 ± 0.05	2.73 ± 0.03

(Mean ± standard deviation).

**Table 5:** DFOV-adjusted PACS measurements with respect to tilt change.

Tilt	*N*	Mean	Standard deviation	Test statistic (*p* value)
Superior 30′ tilt	24	4.62	2.721	234.25^*∗∗∗*^ (0.09)
Superior 24′ tilt	24	4.73	2.715	
Superior 18′ tilt	24	4.78	2.613	
Superior 12′ tilt	24	4.82	2.643	
Superior 6′ tilt	24	4.85	2.721	
Tilt 0	24	4.88	2.752	
Inferior 6′ tilt	24	4.76	2.725	
Inferior 12′ tilt	24	4.71	2.782	
Inferior 18′ tilt	24	4.66	2.811	
Inferior 24′ tilt	24	4.62	2.789	

*N*: total number of measurements. Asterisks denote significant difference between two measurements (^*∗∗∗*^ *p* < 0.001).

**Table 6:** Comparison of DFOV-adjusted PACS measurements and real values.

	Unstandardized coefficients		Standardized coefficients	*t*	*p* value
	*B*	Std. error			
(Constant)	−0.583	0.141		−4.135^*∗∗∗*^	<0.001
FOV	0.045	0.007	0.083	6.322^*∗∗∗*^	<0.001
Tilt 0	0.962	0.013	0.956	73.154^*∗∗∗*^	<0.001
^F^(*p* value)	3797.186^*∗∗∗*^ (<0.001)				
*R* ^2^ (adj. *R* ^2^)	0.997 (0.997)				

Asterisks denote significant difference between two measurements (^*∗∗∗*^ *p* < 0.001).

F: *F*-distribution

*R* ^2^: coefficient of determination.

**Table 7:** *p* value of each measurement interval.

Measurement point	*t*	*F* (*p* value)	*p* value
Hh (horizontal reference line, orbit right – orbit left)	71.451	87111.405 (0.002)	0.009
N (nasion) – A (point A)	71.199	98901.505 (0.002)	0.009
B (basion) – Pg (pogonion)	31.693	25659.497 (0.004)	0.02
P0~zygomatic suture	139.326	571703.871 (0.001)	0.005
Go (gonion) – Me (menton)	410.833	3158113.05 (<0.001)	0.002

**Table 8:** DFOV-adjusted PACS measurements in accordance with tilt change.

Tilt	*N*	Mean	Standard deviation	Test statistic (*p* value)
Superior 30′ tilt	24	4.97	2.795	176.72^*∗∗∗*^ (0.06)
Superior 24′ tilt	24	5.00	2.805	
Superior 18′ tilt	24	5.04	2.816	
Superior 12′ tilt	24	5.07	2.823	
Superior 6′ tilt	24	5.12	2.814	
Tilt 0	24	5.15	2.822	
Inferior 6′ tilt	24	5.03	2.814	
Inferior 12′ tilt	24	5.00	2.805	
Inferior 18′ tilt	24	4.98	2.798	
Inferior 24′ tilt	24	4.96	2.793	

*N*: total number of measurements. Asterisks denote significant difference between two measurements (^*∗∗∗*^ *p* < 0.001).

**Table 9:** DFOV value for each age group, based on analysis of facial CT scans of 200 patients.

Age	DFOV
0~8	15~25
9~15	21~27
16~	25~28

**Table 10:** KSDFOV obtained from DFOV value intervals per age group, based on analysis of 200 facial CTs.

Age	KSDFOV
0~8	22
9~15	24
16~	26

KSDFOV: Korean Standard of DFOV.
